# Evaporation abrupt changes in the Qinghai-Tibet Plateau during the last half-century

**DOI:** 10.1038/s41598-019-56464-1

**Published:** 2019-12-27

**Authors:** Tianci Yao, Hongwei Lu, Wei Feng, Qing Yu

**Affiliations:** 1 Key Laboratory of Water Cycle and Related Land Surface Processes, Institute of Geographic Sciences and Natural Resources Research, Chinese Academy of Sciences, Beijing, 100101 China; 2 University of Chinese Academy of Sciences, Beijing, 100049 China

**Keywords:** Climate sciences, Hydrology

## Abstract

Pan evaporation (*E*_pan_) was regarded as a critical indicator of climate change, especially in the Qinghai-Tibet Plateau (QTP). By using the measured daily *E*_pan_ data of 274 stations in the QTP from 1970 to 2017, the study detected abrupt changes in annual *E*_pan_ series in different spatial scales, through integrating the Mann-Kendall abrupt change test, moving t-test and piecewise linear fitting model. Results showed that abrupt changes existed generally in the QTP where mean and trend abrupt changes were detected in 76.6% and 97.8% of 274 stations during the last half-century. Major abrupt change time of mean values and trends was respectively in around 1996, 1989 and 2007. In comparison, early abrupt changes were observed in the south (south of 30°N) and north (north of 35°N) but late ones in the midland (30–35°N). Corresponding to the low frequent behaviors, pan evaporation paradox only existed in the QTP as a whole in 1970–1990 and was not apparent at site scale, with less than 9.5% of 274 stations detected in different periods. The results confirmed prevailing abrupt change of pan evaporation and its distinct spatial pattern in the QTP.

## Introduction

The Qinghai-Tibet Plateau (QTP), famous as “roof of the world” and “water tower of Asia”, was the highest plateau around the world. It averaged over 4000 meters above sea level, with an area about 200000 square kilometers. Many large rivers originated in the plateau, such as Yangtze, Yellow, Lancang and Nujiang Rivers, supporting more than 1 billion people^1,2^. The QTP had vast territory but sparse population, wherein the influence of human activities was negligible^3^. Because of its vulnerable and sensitive natural environment, the QTP has been considered as the amplifier of global climate change^4–8^ and received increasing attention of worldwide researchers^9^.

Evapotranspiration (*ET*) was both an important component of terrestrial water balance and surface energy balance^10,11^ and an essential force in weather processes and climate patterns^12^. It contributed around 60% to 65% of global precipitation into the atmosphere^11^. Therefore, the study of evapotranspiration change is of great significance to understand climate change and its potential impacts on regional water cycle. Although numerous studies have focused on evapotranspiration-related topics, difficulties remained in accurately measuring and simulating actual evapotranspiration (*ET*_a_), especially at large spatial scale^13^. Overall, data sources commonly used in *ET* scientific community were *in situ* observation (e.g. monitoring of land surface energy flux and pan evaporation), remote sensing observation, model simulation as well as reanalysis datasets^14–18^. Among them, observation data from eddy covariance flux towers were the closest to *ET*_a_, but short of the network limited its popularity^19,20^. As an alternative observation data source, pan evaporation (*E*_pan_) represented potential evaporation from an open water surface under a certain meteorological condition and was often available in long-term time series with good comparability among various regional measurements^21^. It has therefore been widely used in various disciplines such as meteorology, hydrology and ecology^22,23^.

The variation of pan evaporation in the QTP has been studied by some researchers. For example, Liu *et al*.^24^ suggested that annual *E*_pan_ had decreased by 3.06 mm a^−2^ based on 75 stations during 1970–2005, due to combined effects of decreasing wind speed, solar radiation and increasing vapor pressure. Xie *et al*.^25^ reported an overall decreasing rate of 11.91 mm a^−2^ based on 26 stations from 1970 to 2012. Zhang *et al*.^26^ analyzed the spatiotemporal characteristics of annual *E*_pan_ as well as their underlying causes with observations and simulations by PenPan-20 at 77 stations from 1970 to 2011, they found that annual *E*_pan_ existed a striking breakpoint at around 2001 with a significant decreasing trend before 2001 and a remarkable increase afterwards. These studies provided a scientific basis for in-depth understanding of evaporation variations in the QTP. However, the station networks used in above studies were limited, which increased the uncertainty in the identified spatial and temporal patterns. Meanwhile, most studies mainly focused on describing the long-term linear tendency of *E*_pan_ in the entire QTP and its subregions^24,25,27^. Abrupt change was universal and important in the climate system, characterized by the process that climate pattern changed sharply from one stable situation to another^28,29^. For the QTP, unfortunately, it is yet unknown whether *E*_pan_ changes have shifting trend at site scale and differ spatially.

In responses to the above concerns, this study aims to investigate the potential abrupt change characteristics of pan evaporation at site and regional scales by means of several testing methods. Specifically, monthly *E*_pan_ observation data from 274 stations in the QTP during 1970–2017 are firstly obtained and analyzed. Abrupt changes in annual *E*_pan_ series are then detected and tested. The outcomes of this research will advance the understanding of spatiotemporal dynamics of pan evaporation in the QTP, as well as give new insights into the responses of evaporation conditions to climate change.

## Results

### Data preparation

**Figure 1:**
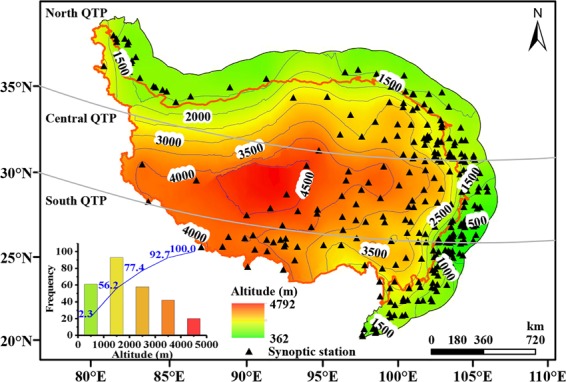
Topography of study area and synoptic stations. Orange line depicted the QTP boundary demarcated by Zhang *et al*.^30^ and the inset showed the frequency of stations in different altitudes. The map was created in ArcMap 10.2, URL: http://www.esrichina-bj.cn/softwareproduct/ArcGIS/, the inset was obtained by origin 9.0, URL: https://www.originlab.com/, and the final figure was generated using Photoshop CC2018, URL: https://onesoftwares.net/.

Considering the continuity in topography change, a 200 km buffer zone away from the QTP boundary demarcated by Zhang *et al*.^30^ was generated, which was the targeted area of this study (Fig. 1). It was further considered as three subregions, i.e. north QTP dominated by the westerlies (north of 35°N), south QTP dominated by the Indian monsoon (south of 30°N) and central QTP influenced by shifting between two circulation systems (30–35°N). The specific climate spatial boundaries and corresponding climatic characteristics can be referred to Yao *et al*.^7^. The observational data used in the study included daily standard Chinese 20 cm diameter pan evaporation (*E*_pan_, mm), daily maximum temperature (*T*_max_, K) and minimum temperature (*T*_min_, K), relative humidity (*RH*, %), sunshine duration (*SD*, h), wind speed and local pressure (*P*, kPa), all of which were taken from a high-quality daily meteorological dataset in the Data Center for Resources and Environmental Sciences, Chinese Academy of Sciences. The dataset and detailed description regarding data quality control were available through http://www.resdc.cn/. Due to its high quality and reliability, the subdataset published in the China Meteorological Data Sharing Service System (http://cdc.cma.gov.cn/) has been widely used in scientific research^31–37^. Though 315 standard synoptic stations were available, only 274 of them were selected during 1970–2017 considering the data quality, with 92, 78 and 104 stations located in the north, south and central QTP, respectively. The station elevations varied within 383.3 m (No. 57401) to 4800 m (No. 55294) (Fig. 1 and Supplementary Table S1).

When ≥25 daily observations were available, a monthly *E*_pan_ was calculated, otherwise it was defined as missing data. According to this criterion, there were 28195 missing data in the selected stations, accounting for 17.9% of the total. PenPan model was then employed to fill the vacancy. Details related to data supplementation were provided in the Supplementary Information file.

### Spatial pattern of annual *E*_pan_

**Figure 2:**
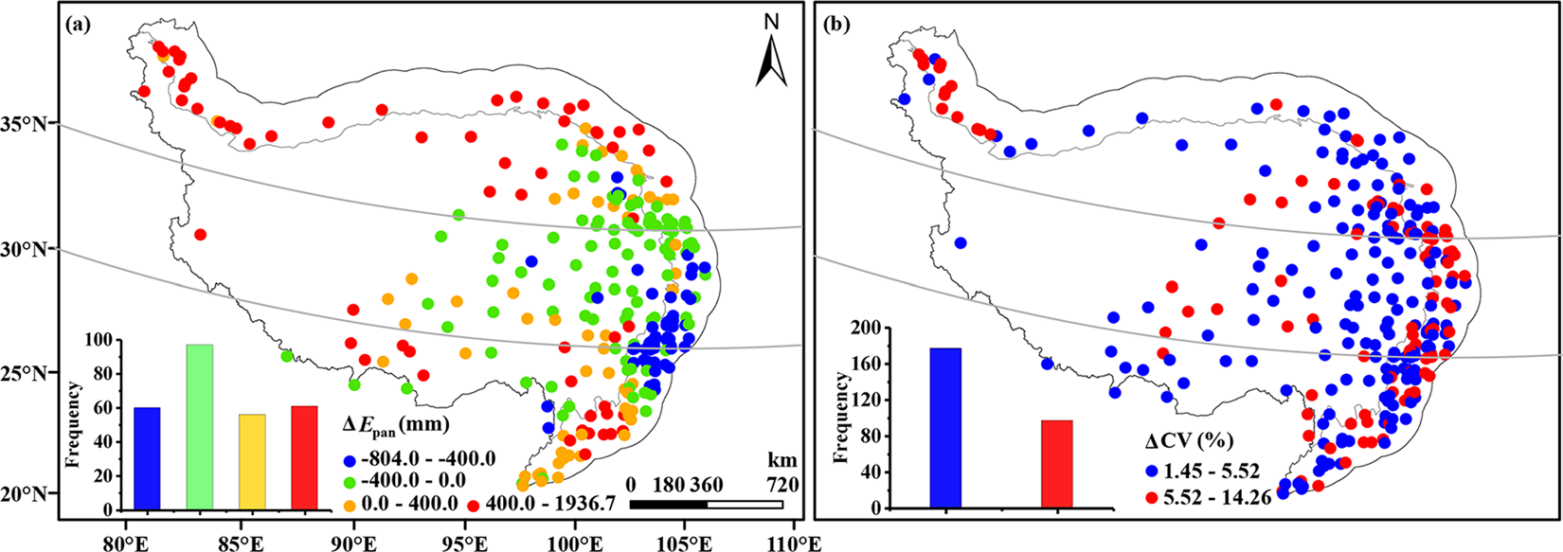
Spatial patterns of the deviations of mean annual values from area-averaged annual *E*_pan_ (**a**). (**b**) same as (**a**) but for coefficients of *E*_pan_ variation. The insets showed the frequency of corresponding deviation values. The maps were created in ArcMap 10.2, URL: http://www.esrichina-bj.cn/softwareproduct/ArcGIS/, the insets were obtained by origin 9.0, URL: https://www.originlab.com/, and the final figure was generated using Photoshop CC2018, URL: https://onesoftwares.net/.

The mean annual *E*_pan_ demonstrated a clear spatial pattern. It was (1639.9 ± 512.8) mm over the study period with a minimum value of 836.7 mm at No. 56278 in the central QTP and a maximum value of 3576.6 mm at No. 52576 in the northern region. To intensively clarify the spatial anomalies of mean annual *E*_pan_ as well as the relationships between the site and area-averaged *E*_pan_, deviations of mean annual *E*_pan_ at each station from area-averaged one were illustrated in Fig. 2(a). It can be found that most stations presented negative deviations, especially in the central and southeastern QTP, suggesting that mean annual *E*_pan_ values in these areas were smaller than that of the entire area; whereas stations located in the north and south QTP owned a positive value, indicating that mean annual *E*_pan_ values at these regions were larger than that of the entire area.

The coefficients of variation (CV) were employed to characterize the inter-annual changes of *E*_pan_ for both the entire QTP and individual stations. In comparison, Fig. 2(b) presented the deviations of CV at each station from that of the entire QTP. It can be found that all deviations of 274 stations were positive and 65% of them varied between 1.45% and 5.52%, indicating that the magnitudes of annual *E*_pan_ changes for individual stations were larger than that of the entire area. Spatially, stations with considerable deviation values were mainly distributed in the edge of the QTP, especially in the northwest and east edges with low elevation.

### Characteristics of abrupt change in annual *E*_pan_

#### Abrupt change of mean value

**Figure 3:**
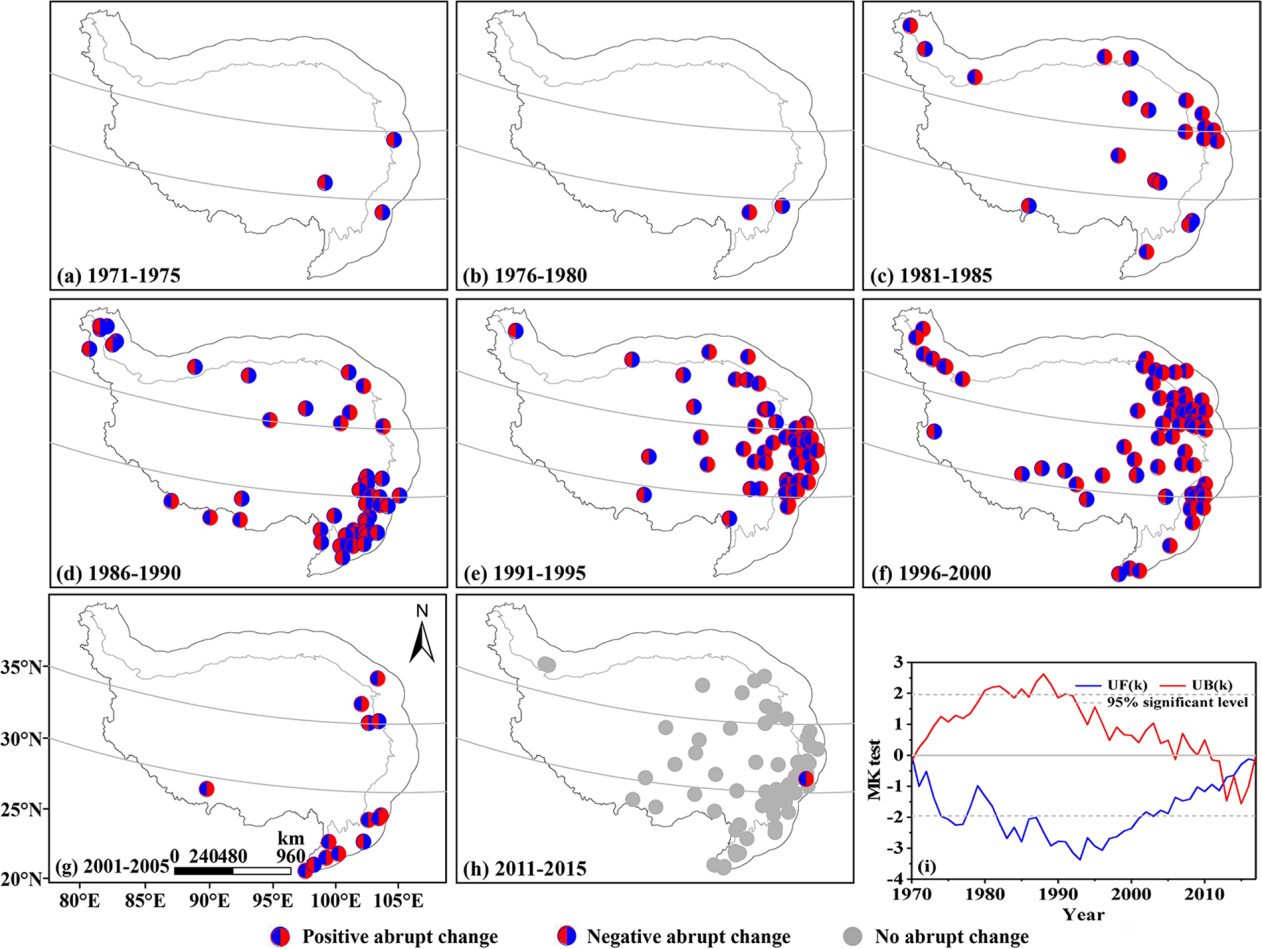
Spatial distributions of mean abrupt changes of annual *E*_pan_ series. (**a**–**h**) abrupt change at site scale, (i) abrupt change in the QTP as a whole. No significant abrupt changes were detected at the period 2006–2010. The maps were created in ArcMap 10.2, URL: http://www.esrichina-bj.cn/softwareproduct/ArcGIS/, the last figure was obtained by origin 9.0, URL: https://www.originlab.com/, and the final figure was generated using Photoshop CC2018, URL: https://onesoftwares.net/.

After detecting the potential mean abrupt changes of annual *E*_pan_ series for 274 stations with Mann-Kendall abrupt change (MK test) and moving t-test methods (*p* < 0.05), the turning points were then illustrated by a 5-year interval in Fig. 3(a–h). Additionally, in order to better grasp the mean abrupt change features, possible mean abrupt changes of area-averaged *E*_pan_ series were also investigated by above methods (Fig. 3(i)).

**Figure 4:**
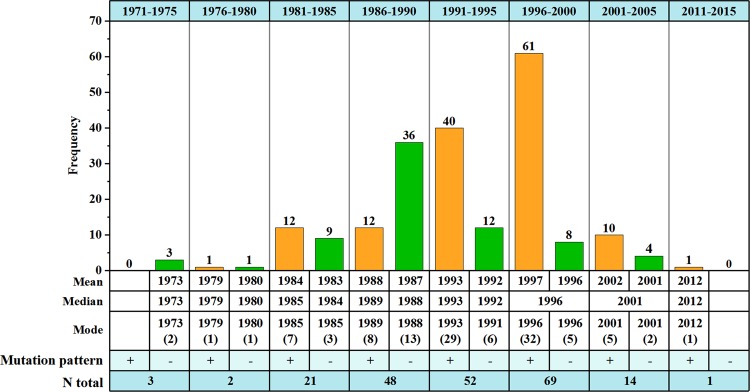
Statistics of mean abrupt changes of annual *E*_pan_ series at site scale. ‘+’ and ‘−’ indicated positive and negative mean abrupt changes, respectively (Same below). The figure was generated by origin 9.0, URL: https://www.originlab.com/.

Abrupt change start time in a certain area was defined as the first year of a consecutive abrupt change process at a 5-year time scale. According to Fig. 4, the annual *E*_pan_ series exhibited mean abrupt change in 76.6% of 274 stations over the study period. In the space domains, earlier mean abrupt change start time was captured in the south QTP, followed by the northern part, with respective start time in 1973 and 1983; whereas the start time in the central QTP was relatively late, especially in the region of 32° to 35°N where it was as late as 1993 (Fig. 3(a–h)). Overall, the mean abrupt changes of annual *E*_pan_ series for each station were primarily detected in 1986–2000, especially around 1996.

The mean abrupt changes were further divided into two types, i.e. positive mean abrupt change where the mean value of subseries after the turning point was greater than that before and negative mean abrupt change which showed a reverse trend to positive mean abrupt change. Combining Figs. 3(a–h) and 4, one can conclude that prior to 1990, the abrupt changes of annual *E*_pan_ series were dominated by negative mean abrupt change with a positive-negative ratio of 25: 49 but turned to 112: 24 after that, suggesting the positive mean abrupt change apparently increased. In general, both the distributions of station numbers displaying positive and negative mean abrupt changes followed the unimodal distribution, with a peak value in 1996 and 1988, respectively. However, both MK and moving t-test indicated no significant abrupt change existed in the area-averaged *E*_pan_ series from a whole perspective (Fig. 3(i)).

#### Abrupt change of trend

**Figure 5:**
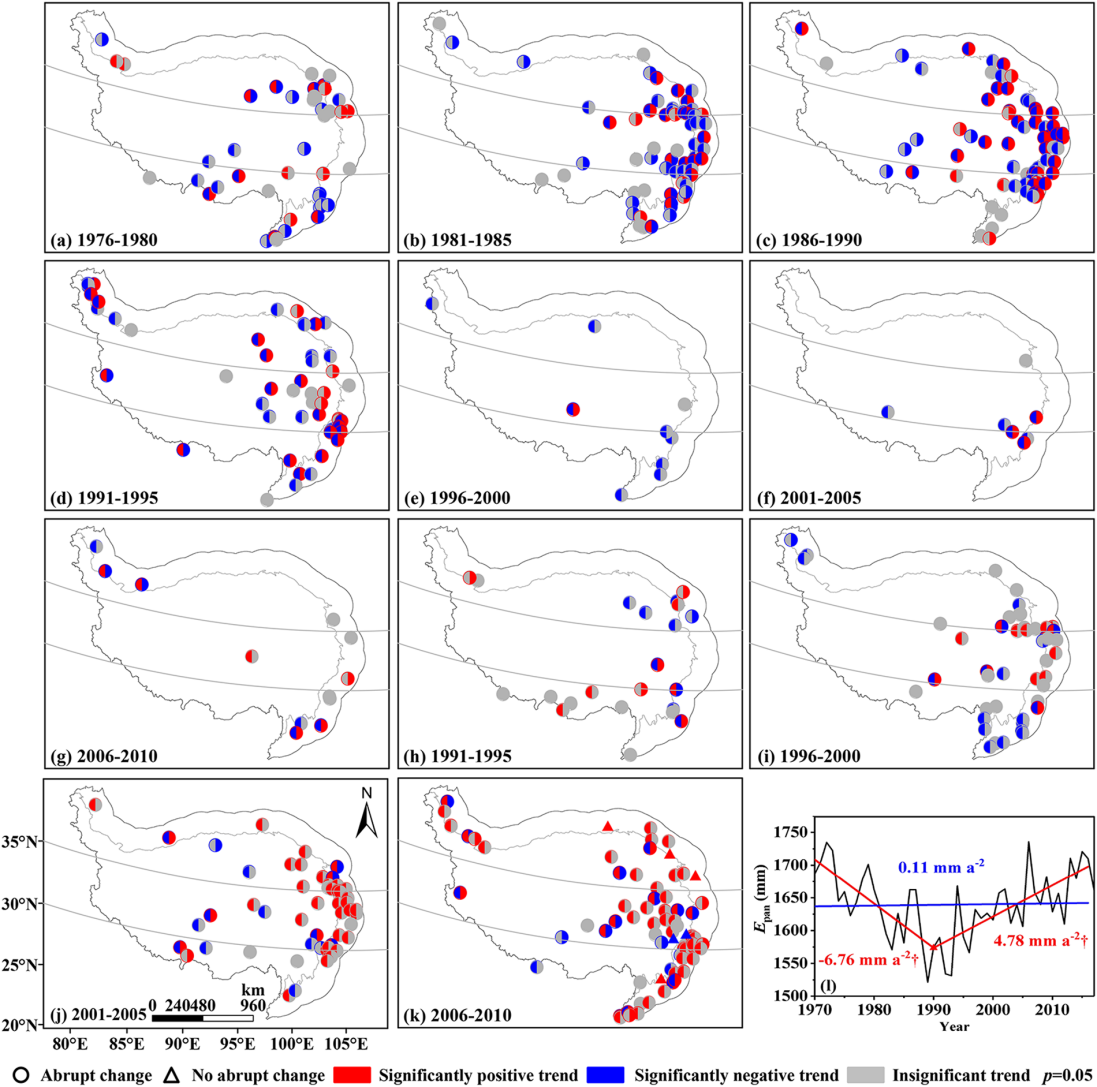
Spatial distributions of trend abrupt changes of annual *E*_pan_ series. (**a–g**) the first abrupt change at site scale, (**h–k**) the second abrupt change at site scale, (l) abrupt change in the QTP as a whole. ‘†’ indicated trend passed the Mann-Kendall’s test at *p* = 0.05 (Same below). The maps were created in ArcMap 10.2, URL: http://www.esrichina-bj.cn/softwareproduct/ArcGIS/, the last figure was obtained by origin 9.0, URL: https://www.originlab.com/, and the final figure was generated using Photoshop CC2018, URL: https://onesoftwares.net/.

Pan evaporation variation was usually nonlinear. By detecting the potential trend abrupt changes of annual *E*_pan_ series for 274 stations using the piecewise linear fitting model (PLFIM), the change points were identified and presented by a 5-year interval in Fig. 5(a–k). Simultaneously, to better understand trend abrupt change features in the QTP, the possible trend abrupt changes of area-averaged *E*_pan_ series were also investigated (Fig. 5(l)).

**Figure 6:**
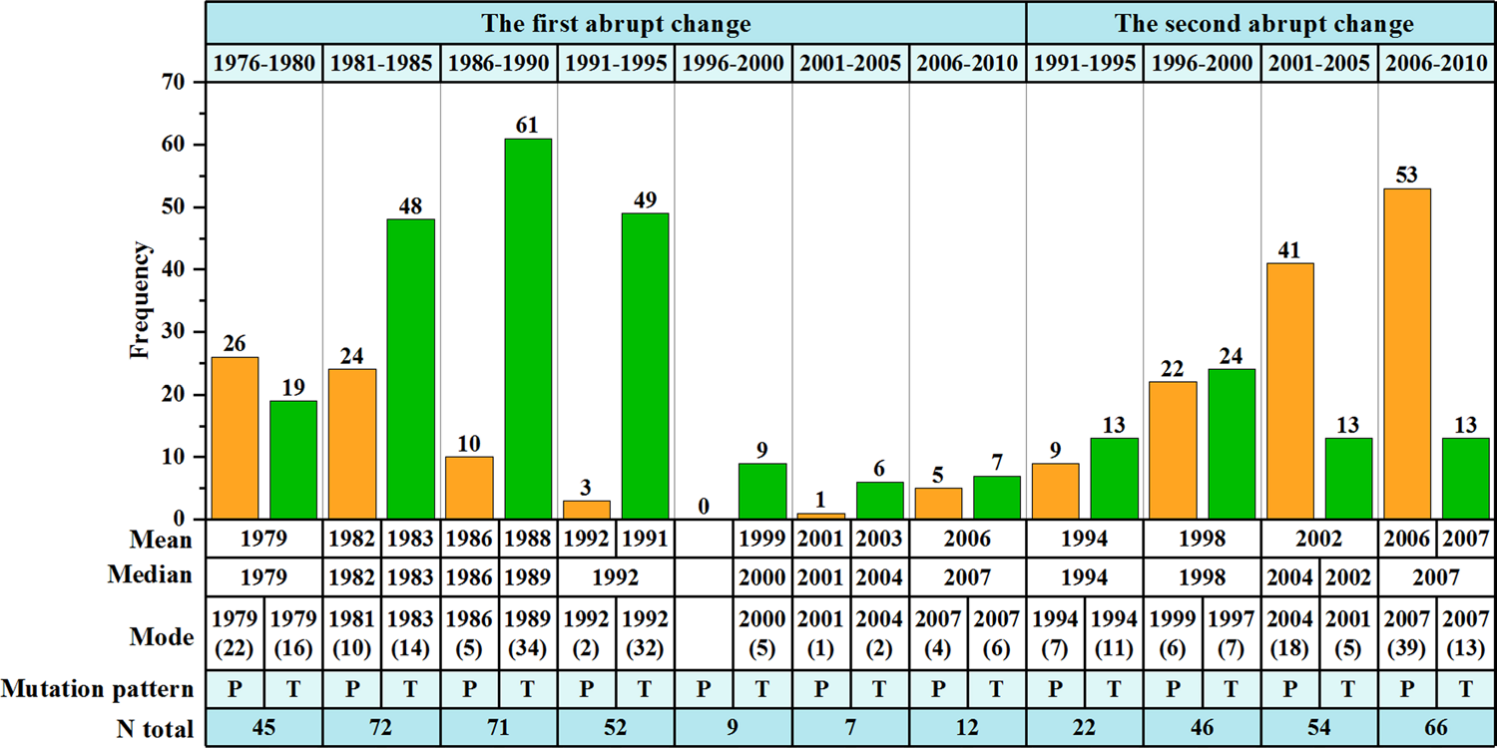
Statistics of trend abrupt changes of annual *E*_pan_ series at site scale. P and T indicated peak and trough abrupt changes, respectively. The figure was generated by origin 9.0, URL: https://www.originlab.com/.

The signals of trend abrupt change were significant widely in station annual *E*_pan_ series. As shown in Fig. 6, 268 out of 274 stations displayed an obviously partial linear trend over the study period, of which 29.9% stations showed only one turning point and 70.1% two turning points. Spatially, for the first turning (Fig. 5(a–g)), the trend abrupt change start time in three subregions was almost synchronous, i.e. about 1979, but that in the region over 32° to 35°N was late until 1983. Similar features can be found for the second turning (Fig. 5(h–k)). Generally, the trend abrupt change of annual *E*_pan_ series occurred primarily in 1981–1995 and 2001–2010, especially in 1989 and 2007.

The trend abrupt changes were also categorized into two types, i.e. peak trend abrupt change where the trend of annual *E*_pan_ series changed from increasing to decreasing through the turning point and trough trend abrupt change where the trend reversed. As shown in Fig. 6, one can easily found out that the trend abrupt change types were remarkably dependent on time. It was specifically dominated by trough trend change prior to 1995 with a peak-trough ratio of 63: 199 and then turned to 116: 50. Interestingly, in spite of different magnitudes for two trend abrupt change types over the study period, both the distributions of station numbers along with abrupt change time displayed clearly temporal patterns. As for peak trend abrupt change, the number of stations decreased gradually in 1970–1995 but increased significantly in 1996–2010, with a maximum value appearing in 1979 and 2007, respectively. A clear unimodal distribution pattern was observed for trough trend change before and after 1995, with the corresponding peak value occurring in 1989 and 1997, respectively. Although trend abrupt changes showed regional diversity, an obvious trough trend abrupt change was depicted in the QTP as a whole, with a join point in 1990 (Fig. 5(l)).

## Discussion

### Spatial pattern of annual *E*_pan_ and its stability

**Figure 7:**
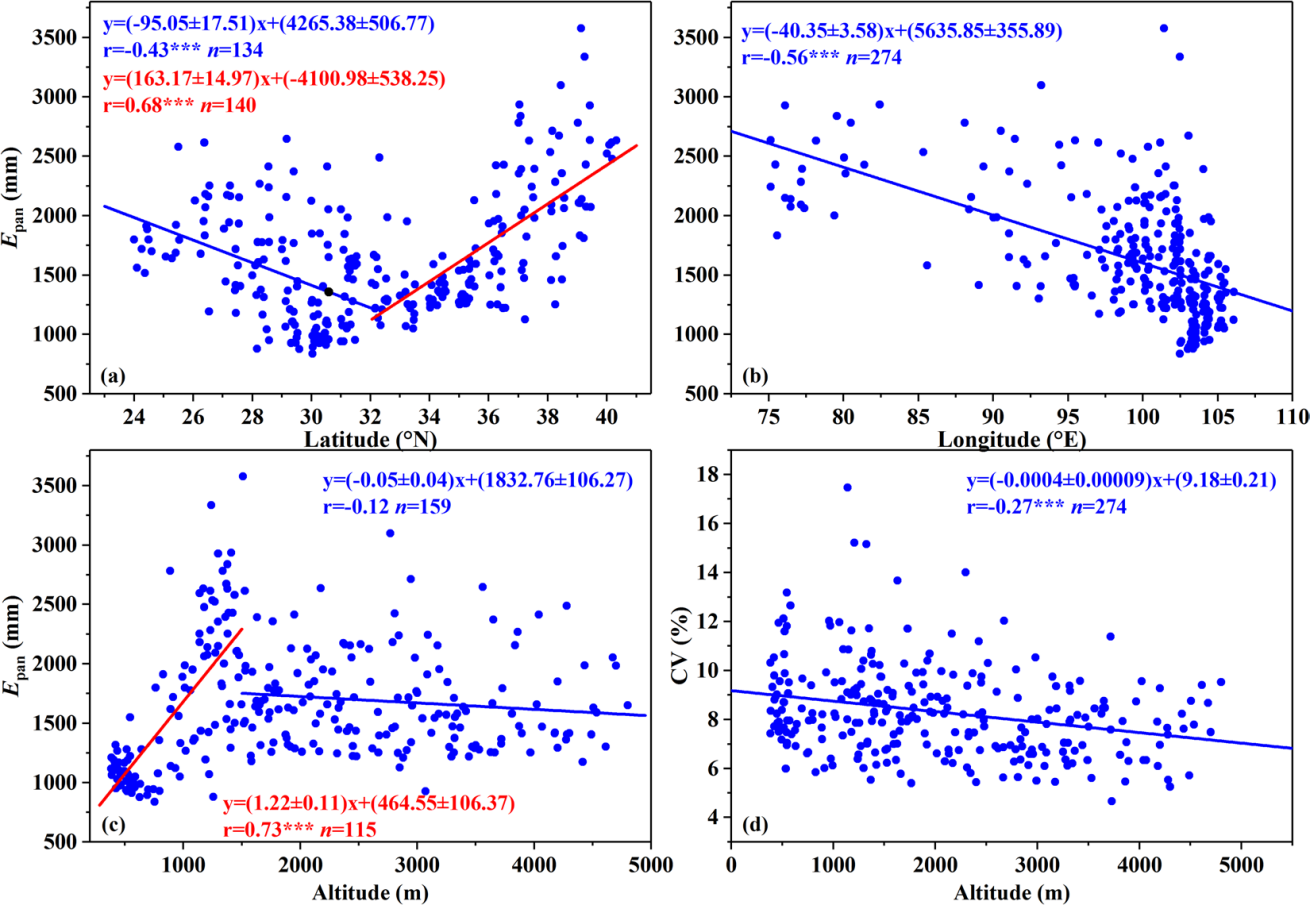
Scatter plots of mean annual values (**a–c**) and coefficients of *E*_pan_ variation (**d**) versus latitude, longitude and altitude at 274 stations, respectively. *, ** and *** indicated correlation passed t-test at *p* < 0.05, *p* < 0.01 and *p* < 0.001, respectively. The figures were generated by origin 9.0, URL: https://www.originlab.com/.

A distinct spatial structure of mean annual *E*_pan_ was uncovered, with large values occurring in the south and north QTP but small ones appearing in the central and southeastern regions. From Fig. 7(a–c), good linear relationships existed between mean annual *E*_pan_ and the corresponding latitude, longitude and altitude. Specifically, mean annual *E*_pan_ decreased by (95.05 ± 17.51) mm with one degree increment of latitude to the south of 32°N but increased by (163.17 ± 14.97) mm to the north of 32°N (Fig. 7(a)); from the west to east, mean annual *E*_pan_ presented a significantly downward trend, with an average lapse rate of (40.35 ± 3.58) mm per one degree increase (Fig. 7(b)). In comparison, significant correlation of mean annual *E*_pan_ with altitude was only detected in a narrow range of 383.3–1500 m, where mean annual *E*_pan_ increased by (122 ± 11) mm for each 100 m increment of altitude (Fig. 7(c)). Considerable low values occurring in the central and southeastern QTP could be attributable to the relatively humid regional atmospheric condition and heavy cloud coverage. The regions were mainly characterized by humid and semi-humid climate, with densely covered river and heavy cloud coverage, particularly in the southeast^38^. Previous studies showed that atmospheric humid, as a crucial aerodynamic factor, exerted directly negative forcing on local atmospheric evaporation demand, while cloud coverage may affect pan evaporation through its influence on global radiation at the earth’s surface. On average, the heavier cloud coverage was, the smaller *E*_pan_ was. Consequently, atmospheric evaporative demands in two regions tended to be at a low level.

Altitudinal dependence of CV was found with a Pearson correlation coefficient of −0.27 (*p* < 0.001, Fig. 7(d)), although the elevation had a poor ability in explaining the observed CV variation. However, the relationships between CV and the corresponding latitude and longitude were insignificant (*p* > 0.1). Generally, the spatial pattern of mean annual *E*_pan_ was similar to Zhang *et al*.^26^ in 1970–2011, although the data series used here was almost 6-year longer. According to the movement trajectory of regional gravity center of annual *E*_pan_ obtained by the Gravity center model, longitude, latitude and altitude coordinates ranged from 97.48° to 98.01°E, 32.72° to 33.03°N and 2019.91 to 2106.43 m, respectively (Supplementary Fig. S2). In addition, trends in three coordinates did not pass the Mann-Kendall’s test at *p* = 0.05. These all indicated the stability of spatial pattern in annual *E*_pan_.

### Prevailing abrupt change of annual *E*_pan_

At site scale, an overall impression of temporal patterns in annual *E*_pan_ series was that the shifting mean values and changing trends could be evidently observed, although the time and station numbers were regionally different. Approaximately 76.6% stations displayed significant mean abrupt changes and nearly all stations (97.8%) showed obviously partial linear trends. Both mean and trend abrupt changes had spatial patterns appearing firstly in the south and north QTP and then to the central region. The latest abrupt change was mainly observed in the north part of the central QTP (32–35°N). Previous studies showed that the north QTP was mainly controlled by the westerlies and the south one dominated by the Indian monsoon. Affected by the transition between the westerlies and monsoon systems, *δ*^18^O in precipitation presented no clear extrema for either winter or summer in the central QTP (32–35°N), with the precipitation and temperature showing small maxima in summer compared to the north and south^7^. This complex atmospheric circulation pattern may be responsible for small regional annual *E*_pan_ and relatively late abrupt change start time in the central region. It should be noted that the above spatial patterns and possible impact factors in different regions, to some extent, could represent the general mechanisms of abrupt changes in annual *E*_pan_ series. However, it might not be applicative for every individual synoptic station in the region. For instance, as shown in Figs. 3 (a–h) and 5(a–k), some nearby stations showed different abrupt changes in the central QTP. It was probably related to the local underlying surface characteristics.

In terms of the area-averaged time series, no significant mean abrupt changes were detected over the study period, but a clear trough trend abrupt change pattern was depicted with a turning point in 1990 when the decreasing trend of annual *E*_pan_ reversed to increasing one. This trend changing pattern was in agreement with the regional study by Zhang *et al*.^26^ and national investigation in China by Liu *et al*.^39^, but its turning point was 11 years and 1 year earlier than that of Zhang *et al*.^26^ and of Liu *et al*.^39^, respectively. In comparison, there were differences in station number and data series length between this study and Zhang *et al*.^26^. The latter presented annual *E*_pan_ data collected from 77 stations in the QTP from 1970 to 2011, and thereby diversifying the abrupt change detected. This also implied the necessity of considerable data with satisfactorily spatial and temporal resolution for the QTP with high spatial heterogeneity in hydrometeorology. Furthermore, it was generally consistent between the trend abrupt change characteristic of annual *E*_pan_ series and that of NDVI during 1982–2006^40^, which supported the results of pan evaporation abrupt change owing to good relationship between *ET*_a_ and NDVI.

### Limited pan evaporation paradox in the QTP

Evaporation was often expected to have an increasing trend in globally warming climate. Lots of observations, however, showed that pan evaporation has been steadily decreasing in many regions over the past several decades, labeled as “pan evaporation paradox”^16,41–45^. In this study, 271 out of 274 stations showed an overall warming trend during the last half-century, and 64.6% of them passed the Mann-Kendall’s test at *p* = 0.05. During the same period, annual *E*_pan_ presented a decreasing trend at 154 out of 274 stations, and 24.0% of them satisfied the Mann-Kendall’s test at the same *p*-value. However, consequential significant pan evaporation paradox, a statistically significant decreasing trend in annual *E*_pan_ corresponding to warming trend (*p* < 0.05), was only observed in 26 stations (Supplementary Fig. S3). By calculating the trends of annual *E*_pan_ of 274 stations during the segmented periods determined by the turning points and the corresponding annual temperature subseries, it was found that pan evaporation paradox only existed in limited stations in three potential periods (Supplementary Fig. S3). Specifically, in the first subperiod (about 1970–1995), 82 out of 126 stations with a warming trend appeared a decreasing trend in annual *E*_pan_, but only 15 stations showed significant pan evaporation paradox; in the second subperiod (about 1996–2007) and third subperiod (after 2008), similarly, 63 and 89 out of 274 stations showed increasing in annual temperature but decreasing in annual *E*_pan_, while only 12 and 2 of them supported the significant pan evaporation paradox, respectively. Therefore, significant pan evaporation paradox scattered in the study periods.

From a regional perspective, the paradox in the QTP as a whole was only observed before 1990, which was consistent with Xing *et al*.^32^ but much different from Liu *et al*.^24^ who used the records of 75 synoptic stations between 1970 and 2005 (Table 1). These findings indicated the necessity of considerable data with satisfactorily spatial and temporal resolution in the QTP. Besides, early studies showed that the paradox just existed in a certain stage or region throughout the world^32,46–48^, indicating that the spatial heterogeneity and time-dependent behavior was a global common phenomenon.

**Table 1:** Comparison of evaporation paradox studies in the QTP.

Region and station number	Study period	Evaporation paradox	Reference
QTP, 274	1970–2017	exist at regional scale in 1970–1990 but not prevail at site scale	This study
QTP, 75	1970–2005	exist at regional scale in 1970–2005	Liu *et al*.^24^
China, 602 QTP, 82	1961–2011	exist at regional scale in 1973–1992	Xing *et al*.^32^
China, 317	1956–2005	exist at national scale but not in northeast and southeast China	Cong *et al*.^46^
China, 599	1960–2013	seasonal, decadal patterns but spatial difference	Huang *et al*.^48^
Global, grid	1980–2011	ENSO dominates the paradox	Miralles *et al*.^47^

In the QTP, therefore, pan evaporation paradox did exist as a whole in 1970–1990 but not prevail at site scale (less than 9.5% of observation stations) in different periods. There was no necessary connection between the paradox and local climate. The limited significant paradox appeared in a few stations was probably caused by random fluctuation of annual *E*_pan_ series and needed further study.

### Abrupt change of *E*_pan_ in different climate zones

**Figure 8:**
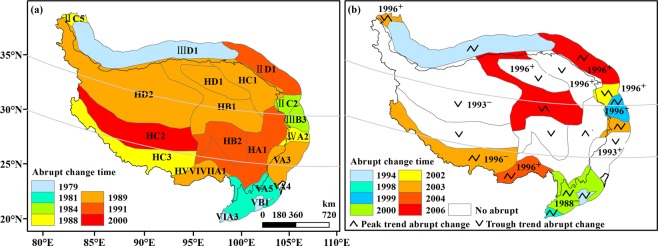
Abrupt changes of zone-averaged annual *E*_pan_ series in different climate zones. (**a**) the first trend abrupt change, (**b**) mean abrupt change and the second trend abrupt change. The shaded patterns and numbers indicated trend and mean abrupt change time, respectively. The maps were generated in ArcMap 10.2, URL: http://www.esrichina-bj.cn/softwareproduct/ArcGIS/.

To clarify the influences of different climate zoning schemes on the abrupt change detection of annual *E*_pan_ series, we further compared the results based on Yao’s zoning^7^ with a more traditional scheme^49^ (Supplementary Table S3) which divided the QTP into 19 climate zones according to spatial-temporal characteristics of temperature and aridity. Figure 8 showed the detection results of abrupt change of zone-averaged annual *E*_pan_ series. From Fig. 8(b), there was prevailing mean abrupt change in *E*_pan_ series (*p* < 0.05), with an exception for climate zones in the central QTP. The mean abrupt changes were dominated by positive abrupt change and mainly occurred in around 1996, but as early as 1988 in VA5 Zone. These were in good agreement with those at site scale.

From Fig. 8(a,b), the trend abrupt change of *E*_pan_ was observed in all climate zones. Specifically, the peak-trough trend abrupt change was dominant in climate zones along the edge of the QTP, while the trough abrupt change dominated in the hinterland. After the last trend abrupt change, *E*_pan_ exhibited an upward trend in most climate zones (Fig. 8(b)). By comparison, the trend abrupt change time in the south and north QTP was generally earlier than that in the central, which were broadly similar to the results obtained from the climate zoning scheme with three subregions.

### Abrupt change of annual actual evapotranspiration

**Figure 9:**
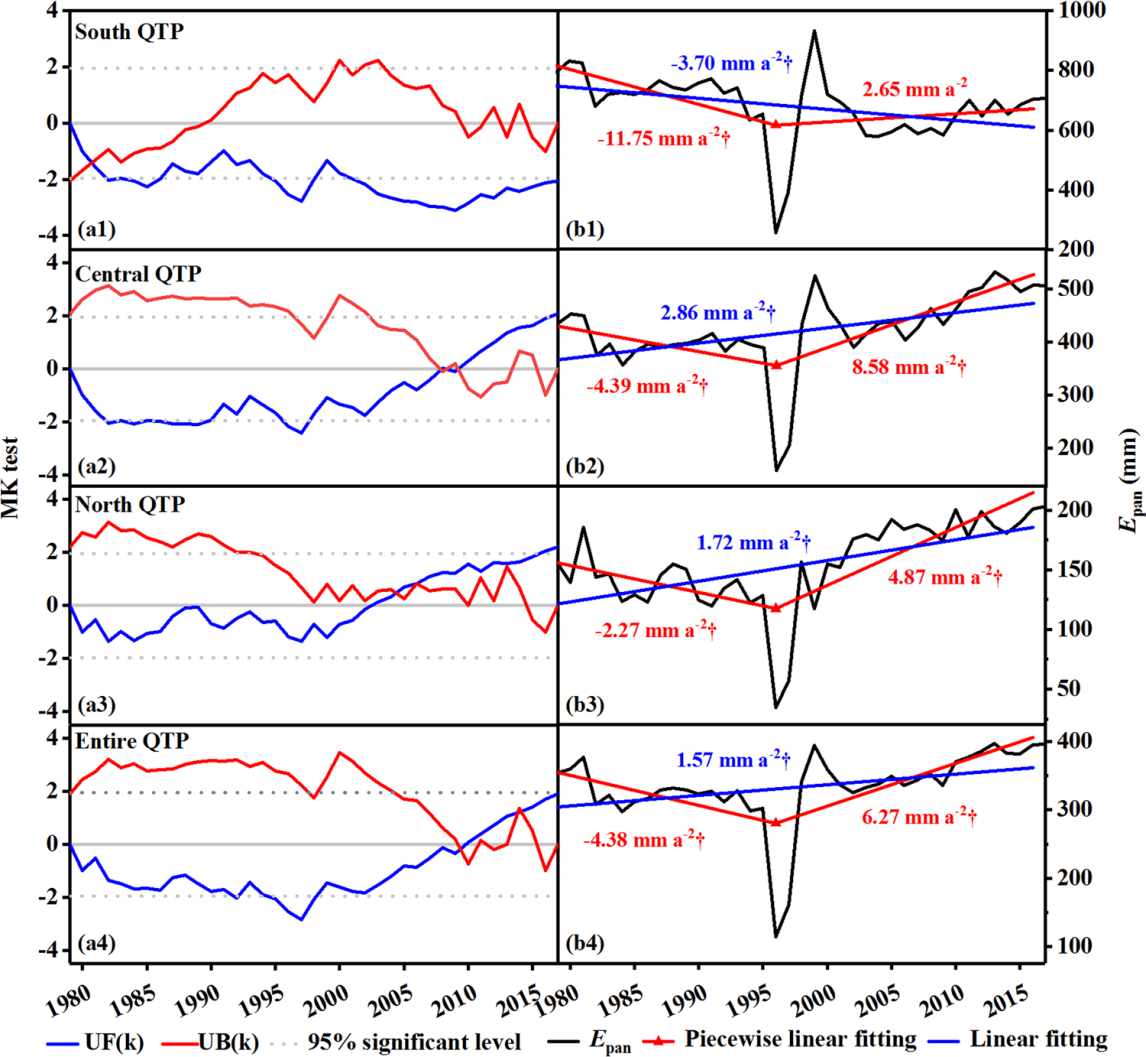
Abrupt changes of annual actual evapotranspiration series in the QTP and three subregions. (**a1–a4**) mean abrupt change, (**b1–b4**) trend abrupt change. The figures were generated by origin 9.0, URL: https://www.originlab.com/.

Monthly actual evapotranspiration from the Global Land Data Assimilation System (GLDAS, https://disc.gsfc.nasa.gov/) was used to make comparison with observation dataset. Figure 9 showed the results of abrupt changes in annual *ET*_a_ series for the entire QTP and three subregions. A distinct spatial pattern of mean abrupt change was found, where the mean abrupt change time of annual *ET*_a_ was the earliest in the south QTP (around 1981), followed by the north QTP (around 2004) and central QTP (around 2009, Fig. 9(a1–a3)). Although the two curves in Fig. 9(a4) intersected twice within the significance lines of *p* = 0.05, the abrupt change was insignificant according to moving t-test at *p* = 0.05, indicating that no mean abrupt change appeared with regard to the QTP as a whole.

In term of changing trend, all three subregions presented substantially trough trend abrupt changes with a common join point around 1997 (Fig. 9(b1–b3)). Further, area-averaged annual *ET*_a_ tended to decrease before 1997, and then increase significantly afterwards, suggesting that substantial trend abrupt change existed (Fig. 9(b4)). In comparison, the trend abrupt change of area-averaged annual *ET*_a_ appeared around 7 years later than that of annual pan evaporation, because of the delayed response of annual *ET*_a_ to the change in atmospheric evaporation conditions. These results further supported the findings from observation dataset.

## Conclusions

In this study, we modified PenPan model to estimate the missing monthly *E*_pan_ data at 274 stations in the QTP during 1970–2017, and potential evaporation abrupt changes were then detected. Mean annual *E*_pan_ displayed spatial heterogeneity with high values in the south and north QTP while low in the central and southeastern regions, and the spatial distribution of mean annual *E*_pan_ showed stability during the last half-century. In addition, mean and trend abrupt changes generally existed in 76.6% and 97.8% of total stations. The major abrupt change time of mean values and trends was respectively at 1996, 1989 and 2007 in the order of south-north-central. It was relatively late in the midland, especially in the region of 32° to 35°N. Pan evaporation paradox did not prevail (less than 9.5%) in the QTP at site scale and only existed in 1970–1990 at regional scale. The findings presented valuable monthly *E*_pan_ dataset for the relevant researches, and further advanced our understanding of evaporation response to climate and hydrological cycle changes. Future studies may focus on the abrupt change mechanism through modeling techniques based on high-resolution and long-term datasets.

## Methods

### PenPan model

PenPan model was a physically-based model for estimating monthly pan evaporation^50^, which coupled the Linacre’s Penpan model^51^, Pereira’s Penman-Monteith-style model^52^ and Thom’s Penman-style model^53^. In comparison with the mathematical-statistical models, e.g. multiple linear regression model, it showed better performance due to integrating the influences of local environmental factors and the structure of pan itself^50^. In this model, monthly *E*_pan_ (mm) was subdivided into radiative (*E*_rad_, mm s^−1^) and aerodynamic components (*E*_aero_, mm s^−1^)^31^:

1 $${E}_{{\rm{pan}}}={E}_{{\rm{rad}}}+{E}_{{\rm{aero}}}=\frac{\Delta {R}_{{\rm{n}}}}{(\Delta +\alpha \gamma )\lambda }+\frac{\alpha \gamma f({U}_{2})VPD}{\Delta +\alpha \gamma }$$

where Δ, with a unit of Pa·K^−1^, denoted the slope of saturation vapor pressure (*e*_s_) curve at a given air temperature at 2 m above the ground level (*T*_a_, K), *T*_a_ for monthly periods was defined as the mean of corresponding *T*_max_ and *T*_min_, *R*_n_ was net radiation of the pan (W·m^−2^), *α* refered to the ratio of the effective surface area for the transmission of heat and water vapor (=5)^54^, 𝛾 was the psychrometric constant (≈ 67 Pa·K^−1^), 𝜆 indicated the latent heat of vaporization of water (=2.45 × 10^6^ J·kg^−1^), *U*_2_ showed the mean wind speed at 2 m above the ground level (m·s^−1^), *VPD* was the vapor pressure deficit at 2 m above the ground level, and *f*(*U*_2_) was the wind function for transfer of water vapor at 2 m above the ground level (kg·m^−2^·s^−1^·Pa^−1^). The specific calculation formulas for each parameter in the model were shown as follows:

2 $$\Delta =611\frac{\lambda {M}_{{\rm{w}}}}{R{T}_{{\rm{a}}}^{2}}\times \exp \,[\frac{17.27({T}_{{\rm{a}}}-273.15)}{{T}_{{\rm{a}}}-36.15}]$$

where *M*_w_ was the molecular mass of water (=0.018 kg mol^−1^), *R* was the ideal gas constant (=8.314 J mol^−1^ K^−1^). The *R*_n_ was estimated using an empirical equation recommended by FAO^55^:

3 $${R}_{{\rm{n}}}=(1-{A}_{{\rm{p}}}){R}_{{\rm{sp}}}+{R}_{{\rm{nl}}}$$

where *R*_sp_ was incoming shortwave irradiance of the pan, including direct shortwave irradiance and the additional interception by pan walls^50^, *A*_p_ denoted the albedo for pan (=0.14), *R*_nl_ showed net long-wave irradiance. *R*_sp_ was estimated using the method described by Yang *et al*.^54^:

4 $${R}_{{\rm{sp}}}=({f}_{{\rm{dir}}}({p}_{{\rm{rad}}}-2)+2+2a)\times {R}_{{\rm{s}}}$$

5 $${P}_{{\rm{rad}}}=1.70+3\times {10}^{-4}{\varphi }^{2}$$

where *f*_dir_ was the fraction of direct radiation in global solar radiation, *P*_rad_ indicated the pan radiation factor, *φ* was the absolute value of latitude (degrees). The land surface albedo was *a* (=0.23), which was defined as a value appropriate for short and green grass. Wang *et al*.^38^ established the regression equations as follows, which successfully estimated the monthly diffuse radiation in China at site scale:

6 $${R}_{d}/{R}_{s}=(1.02\pm 0.08)+(\,-\,1.13\pm 0.17){R}_{s}/{R}_{a}\,{\rm{for}}\,{\rm{the}}\,{\rm{southern}}\,{\rm{region}}$$

7 $${R}_{d}=(0.20\pm 1.13)+(\,-\,1.13\pm 0.17){R}_{s}\,{\rm{for}}\,{\rm{the}}\,{\rm{northern}}\,{\rm{region}}$$

Generally, the values of monthly *R*_d_ at stations in the QTP can be estimated well by both regression methods. Given that most synoptic stations distributed in the southern region, Eq. (6) was employed to calculate *f*_dir_. *R*_a_ refered to extraterrestrial solar radiation (MJ m^−2^ day^−1^), *R*_s_ was global solar radiation (MJ m^−2^ day^−1^) and can be calculated by:

8 $${R}_{{\rm{s}}}=({a}_{{\rm{s}}}+{b}_{{\rm{s}}}\frac{n}{N}){R}_{{\rm{a}}}$$

The monthly mean Angstrom coefficients, i.e. *a*_s_ and *b*_s_, calculated by Yin *et al*.^56^ were employed in this study. The *R*_nl_ was calculated as follows^55^:

9 $${R}_{{\rm{nl}}}=0.5\sigma ({T}_{{\rm{\max }}}^{4}+{T}_{{\rm{\min }}}^{4})(0.34-0.14\sqrt{{e}_{{\rm{a}}}})(1.35{f}_{{\rm{dir}}}-0.35)$$

where *σ* was the Stefan-Boltzmann constant (4.903*10^−3^ J K^−4^ m^−2^ day^−1^), *T*_max_ and *T*_min_ denoted the maximum and minimum temperature (K) during the 24 h-period, respectively. Zhang *et al*.^26^ reported that the following regression approaches can well characterize the transfer process of water vapor over the QTP:

10 $$f({U}_{2})=1.39\times {10}^{-8}(1+1.35{U}_{2})$$

11 $${U}_{2}=4.87{U}_{10}/\mathrm{ln}(678-5.42)$$

where *U*_10_ showed the mean wind speed at 10 m above the ground level (m·s^−1^).

*VPD* can be calculated as follows:

12 $$VPD=4098\,(610.8\,\exp (\frac{17.27{T}_{{\rm{a}}}}{{T}_{{\rm{a}}}+237.3}))/{({T}_{{\rm{a}}}+237.3)}^{2}$$

### Trend free pre-whitening

The autoregressive process had an obvious influence on the results obtained by nonparametric test methods (e.g. Mann-Kendall rank statistical test), especially the lag-1 autoregressive process^57,58^. Therefore, in this study, the lag-1 autocorrelation coefficient of annual *E*_pan_ data was firstly calculated and tested prior to applying Mann-Kendall rank statistical test to assess the significance of trends and to detect the abrupt changes in annual *E*_pan_ series. Results showed that 235 out of 274 stations exhibited a significant autocorrelation with Student’s t-test (t-test) at *p* = 0.05, varying from 0.29 to 0.83. A trend free pre-whitening technique was then applied to eliminate the serial correlation^57^. The new lag-1 autocorrelation values fell in (−0.23, 0.22) and none of them passed the t-test at *p* = 0.10, indicating that new time series was not influenced by the serial correlation.

### Abrupt change analysis

Mann-Kendall abrupt change test was a commonly used nonparametric test method for abrupt changes of mean values in hydrometeorological time series, but it showed uncertainty for time series with two or more abrupt change points. Hence, moving t-test, a parametric test method, was also applied to verify the abrupt change. The procedures of MK and moving t-test can be found in Zhao *et al*.^59^ and not provided here. Meanwhile, the piecewise linear fitting model proposed by Tomé *et al*.^60^ was selected to detect the turning point of annual *E*_pan_ trend. PLFIM utilized a least-square approach to compute the best combination of continued straight lines fitting a given time series, subject to a set of constraints on the minimum distance between neighboring breakpoints and on the minimum trend change before and after the breakpoint. According to early studies^60–62^ and considering the length of annual *E*_pan_ series, we set the constraints as follows: a minimum 15-year interval between neighboring breakpoints, a minimum 10-year length for the first and last segments and an opposite sign between two consecutive change trends. Further, once turning points were detected, the Mann-Kendall trend analysis method was adopted to assess the significance of change during the segmented period^58^.

### Gravity center model

The movement trajectory of regional gravity center of annual *E*_pan_ was examined in this study by using the following equations^63^:

13 $${\overline{x}}_{{\rm{t}}}=\mathop{\sum }\limits_{i=1}^{i=274}{E}_{{\rm{pan}}\_{\rm{ti}}}{x}_{{\rm{i}}}/\mathop{\sum }\limits_{i=1}^{i=274}{E}_{{\rm{pan}}\_{\rm{ti}}}$$

14 $${\overline{y}}_{{\rm{t}}}=\mathop{\sum }\limits_{i=1}^{i=274}{E}_{{\rm{pan}}\_{\rm{ti}}}\,{y}_{{\rm{i}}}/\mathop{\sum }\limits_{i=1}^{i=274}{E}_{{\rm{pan}}\_{\rm{ti}}}$$

15 $${\overline{z}}_{{\rm{t}}}=\mathop{\sum }\limits_{i=1}^{i=274}{E}_{{\rm{pan}}\_{\rm{ti}}}{z}_{{\rm{i}}}/\mathop{\sum }\limits_{i=1}^{i=274}{E}_{{\rm{pan}}\_{\rm{ti}}}$$

where $\bar{{\rm{x}}}\,$_t_, $\bar{{\rm{y}}}\,$_t_ and $\bar{{\rm{z}}}\,$_t_ respectively denoted the latitude, longitude and altitude coordinates of regional gravity center of annual *E*_pan_ at year *t*, *E*_pan,ti_ was annual *E*_pan_ at year *t* for stat*i*on *i*, (*x*_i_*, y*_i_*, z*_i_) represented the corresponding coordinates of stat*i*on *i*.

## Supplementary information

Supplementary Information

## Data Availability

Daily meteorological dataset used in this study is available in the Data Center for Resources and Environmental Sciences, Chinese Academy of Sciences (http://www.resdc.cn). Monthly actual evapotranspiration and temperature datasets from the Global Land Data Assimilation System (GLDAS) are available from GES DISC Web site (https://disc.gsfc.nasa.gov/). The monthly and annual pan evaporation data are obtained from the corresponding author on reasonable request.
