# Supplementary material for: Evaporation abrupt changes in the Qinghai-Tibet Plateau during the last half-century
Source: Sci Rep. 2019 Dec 27;9:20181. doi: 10.1038/s41598-019-56464-1 (PMC6934787; doi:10.1038/s41598-019-56464-1)
Supplement: Supplementary file 1 — Supplementary Information [file 41598_2019_56464_MOESM1_ESM.pdf]

# Evaporation abrupt changes in the Qinghai-Tibet Plateau during the last half-century

Tianci Yao[1,2], Hongwei Lu[1*], Wei Feng[1,2], Qing Yu[1,2]

[1]*Key Laboratory of Water Cycle and Related Land Surface Processes, Institute of Geographic Sciences and Natural Resources Research, Chinese Academy of Sciences, Beijing 100101, China*

[2]*University of Chinese Academy of Sciences, Beijing 100049, China*

*Correspondence and requests for materials should be addressed to H.L. (email: luhw@igsnrr.ac.cn)

# Supplementary Information Text

## Evaluation of $E_{pan}$ simulation

Several evaluation metrics, including the linear regression equation and its determination coefficient ($R^2$), root mean square error (RMSE) as well as the difference of mean monthly values (MD) between the simulated ($E_{pan\_simu}$) and observed $E_{pan}$ ($E_{pan\_obs}$), were calculated to assess the performance of PenPan model at a monthly time scale (Supplementary Fig. S1 and Table S2). Generally, the agreement between $E_{pan\_simu}$ and $E_{pan\_obs}$ was satisfactory. As shown in Supplementary Fig. S1, the regression slope between $E_{pan\_simu}$ and $E_{pan\_obs}$ of all stations was 0.96, and the corresponding $R^2$, RMSE and MD were 0.96, 16.94 mm and 0.00 mm, respectively. The average value of regression slopes at different stations was 0.92 ± 0.05 (mean ± 1 s.d.), and the corresponding average values of $R^2$ and RMSE were 0.96 ± 0.03 and (15.92 ± 5.11) mm, respectively (Supplementary Table S2). Additionally, a negligible MD between $E_{pan\_simu}$ and $E_{pan\_obs}$ presented at 274 stations. These metrics indicated that PenPan model was applicable to estimate monthly $E_{pan}$ in the highlands at both site and regional scales.

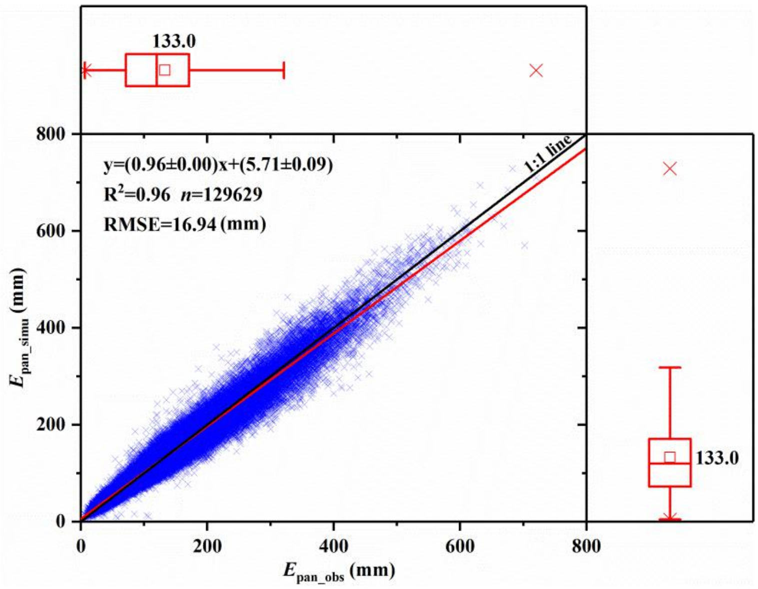


Figure S1. Comparison of simulated and observed monthly $E_{pan.}$ Boxplots showed minima (cross), 25th percentile, median (solid line), average (square), 75th percentile and maxima (cross). The figure was generated by origin 9.0, URL: https://www.originlab.com/.

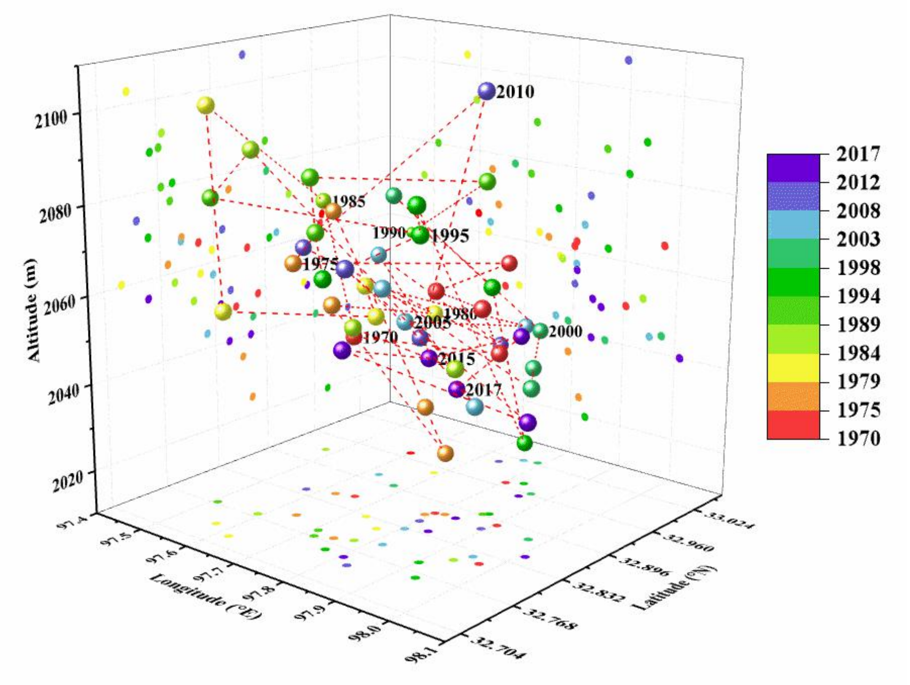


Figure S2. Movement trajectory of regional gravity center of annual $E_{\mathrm{pan}}$ during 1970-2017.

The figure was generated by origin 9.0, URL: https://www.originlab.com/.

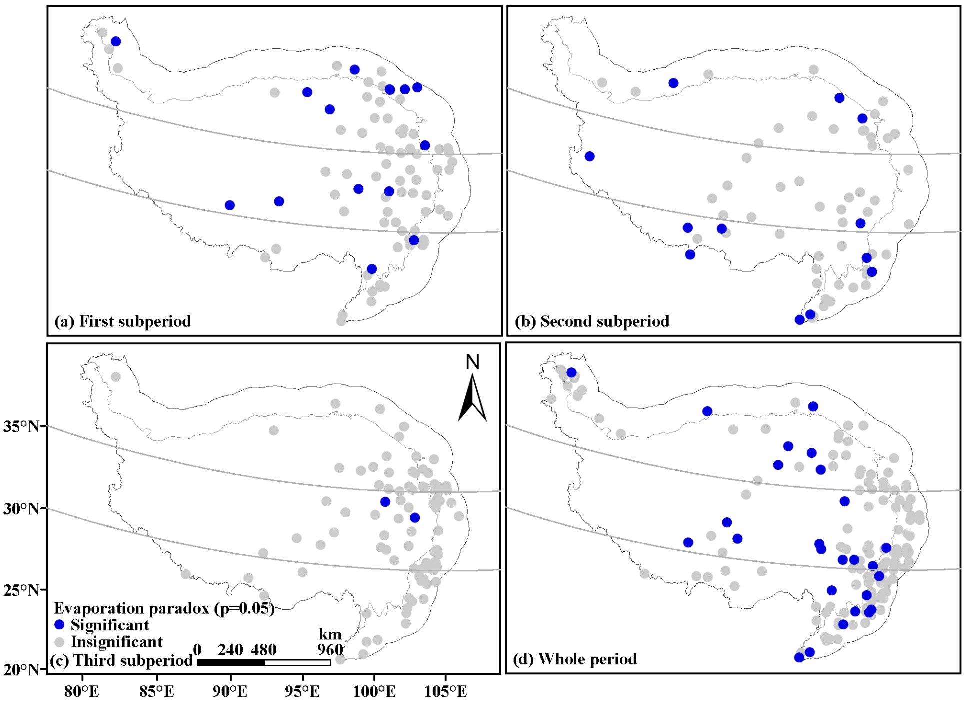


Figure S3. Spatial distributions of pan evaporation paradox in the whole and three-segmented periods. The maps were generated in ArcMap 10.2, URL: http://www.esrichina-bj.cn/softwareproduct/ArcGIS/.

Table S1. Basic information about 274 stations.

| WMO ID | Latitude (°N) | Longitude (°E) | Altitude (m) | WMO ID | Latitude (°N) | Longitude (°E) | Altitude (m) |
|---|---|---|---|---|---|---|---|
| 51804 | 37.46 | 75.14 | 3090.1 | 52652 | 39.05 | 100.17 | 1461.1 |
| 51886 | 38.15 | 90.51 | 2944.8 | 52661 | 38.48 | 101.05 | 1765.5 |
| 52533 | 39.46 | 98.29 | 1477.2 | 52674 | 38.13 | 101.56 | 2093.9 |
| 52602 | 38.45 | 93.20 | 2770.0 | 52681 | 38.38 | 103.05 | 1367.5 |
| 52633 | 38.49 | 98.25 | 3367.0 | 52797 | 37.11 | 104.03 | 1630.9 |
| 52643 | 38.50 | 99.37 | 2311.8 | 52884 | 36.21 | 103.57 | 1668.5 |
| 52645 | 38.26 | 99.36 | 3314.0 | 52885 | 36.45 | 103.15 | 2118.8 |
| 52656 | 38.28 | 100.49 | 2232.0 | 52889 | 36.03 | 103.53 | 1517.2 |
| 52657 | 38.11 | 100.15 | 2787.4 | 52895 | 36.34 | 104.41 | 1398.2 |
| 52679 | 37.55 | 102.40 | 1531.5 | 52896 | 36.33 | 104.09 | 1736.7 |
| 52713 | 37.51 | 95.21 | 3173.2 | 52980 | 35.58 | 103.18 | 1648.0 |
| 52737 | 37.22 | 97.23 | 2981.5 | 52981 | 35.41 | 103.23 | 2421.0 |
| 52745 | 37.18 | 99.01 | 3417.1 | 52982 | 35.29 | 103.33 | 1953.2 |
| 52754 | 37.20 | 100.08 | 3301.5 | 52983 | 35.52 | 104.09 | 1874.4 |
| 52765 | 37.23 | 101.37 | 2850.0 | 52984 | 35.35 | 103.11 | 1917.2 |
| 52784 | 37.28 | 102.54 | 2072.4 | 52986 | 35.22 | 103.52 | 1893.8 |
| 52787 | 37.12 | 102.52 | 3045.1 | 52993 | 35.41 | 105.04 | 1739.4 |
| 52818 | 36.25 | 94.55 | 2807.6 | 52995 | 35.35 | 104.37 | 1897.2 |
| 52825 | 36.26 | 96.26 | 2790.4 | 52996 | 35.23 | 105.01 | 2450.6 |
| 52836 | 36.18 | 98.06 | 3189.0 | 52998 | 35.08 | 104.12 | 2111.0 |
| 52842 | 36.47 | 99.05 | 3087.6 | 53908 | 35.13 | 105.14 | 1769.8 |
| 52855 | 36.41 | 101.15 | 2675.0 | 55655 | 28.11 | 85.58 | 3810.0 |
| 52856 | 36.16 | 100.37 | 2835.0 | 55690 | 27.59 | 91.57 | 4280.3 |
| 52862 | 36.57 | 101.41 | 2450.0 | 56091 | 34.51 | 104.28 | 1883.3 |
| 52863 | 36.49 | 101.57 | 2480.0 | 56092 | 35.00 | 104.39 | 1728.2 |
| 52866 | 36.44 | 101.45 | 2295.2 | 56093 | 34.26 | 104.01 | 2315.0 |
| 52868 | 36.01 | 101.22 | 2273.0 | 56095 | 34.02 | 104.23 | 1753.2 |
| 52869 | 36.30 | 101.35 | 2667.5 | 56096 | 33.24 | 104.55 | 1079.1 |
| 52874 | 36.29 | 102.25 | 2021.0 | 56181 | 30.41 | 103.42 | 541.7 |
| 52876 | 36.20 | 102.50 | 1813.9 | 56186 | 31.20 | 104.12 | 589.0 |
| 52877 | 36.06 | 102.15 | 2834.7 | 56187 | 30.45 | 103.52 | 547.7 |
| 52908 | 35.13 | 93.05 | 4612.2 | 56188 | 31.00 | 103.40 | 698.5 |
| 52943 | 35.35 | 99.59 | 3323.0 | 56189 | 30.59 | 103.56 | 581.7 |
| 52955 | 35.35 | 100.44 | 3120.0 | 56190 | 31.33 | 104.33 | 519.4 |
| 52957 | 35.15 | 100.36 | 3148.2 | 56192 | 32.57 | 104.40 | 1014.3 |
| 52963 | 35.56 | 102.01 | 2085.7 | 56193 | 32.25 | 104.31 | 893.2 |
| 52968 | 35.02 | 101.28 | 3662.8 | 56195 | 31.48 | 104.44 | 543.1 |
| 52972 | 35.51 | 102.27 | 1921.0 | 56196 | 31.27 | 104.44 | 522.7 |
| 52974 | 35.33 | 102.02 | 2475.0 | 56197 | 31.09 | 104.10 | 535.7 |
| 52978 | 35.11 | 102.30 | 2948.3 | 56198 | 31.19 | 104.30 | 525.7 |
| 52985 | 35.25 | 103.20 | 2162.8 | 56199 | 31.02 | 104.41 | 423.5 |

| | | | | | | | |
|---|---|---|---|---|---|---|---|
| 52988 | 35.22 | 103.43 | 2015.8 | 56272 | 30.49 | 103.53 | 558.5 |
| 55228 | 32.30 | 80.05 | 4278.6 | 56273 | 30.23 | 102.49 | 1022.0 |
| 55279 | 31.23 | 90.01 | 4700.0 | 56276 | 30.27 | 103.49 | 467.0 |
| 55294 | 32.21 | 91.06 | 4800.0 | 56278 | 30.04 | 102.47 | 755.8 |
| 55299 | 31.29 | 92.04 | 4507.0 | 56279 | 30.10 | 102.57 | 744.1 |
| 55472 | 30.57 | 88.38 | 4672.0 | 56280 | 30.05 | 103.07 | 691.3 |
| 55493 | 30.29 | 91.06 | 4200.0 | 56281 | 30.12 | 103.29 | 510.8 |
| 55578 | 29.15 | 88.53 | 3836.0 | 56284 | 30.27 | 103.26 | 518.5 |
| 55591 | 29.40 | 91.08 | 3648.9 | 56285 | 30.34 | 103.28 | 544.7 |
| 55598 | 29.16 | 91.46 | 3560.0 | 56287 | 29.59 | 103.00 | 627.6 |
| 55680 | 28.55 | 89.36 | 4040.0 | 56289 | 30.12 | 103.52 | 437.0 |
| 55681 | 28.58 | 90.24 | 4431.7 | 56290 | 30.47 | 104.11 | 514.5 |
| 55696 | 28.25 | 92.28 | 3860.0 | 56291 | 30.56 | 104.17 | 469.0 |
| 55773 | 27.44 | 89.05 | 4300.0 | 56295 | 30.23 | 104.33 | 448.5 |
| 56004 | 34.13 | 92.26 | 4533.1 | 56296 | 30.49 | 104.26 | 493.5 |
| 56016 | 33.51 | 95.37 | 4179.1 | 56297 | 30.01 | 104.09 | 436.5 |
| 56018 | 32.53 | 95.17 | 4066.4 | 56298 | 30.08 | 104.36 | 417.0 |
| 56021 | 34.07 | 95.48 | 4175.0 | 56371 | 29.53 | 102.13 | 1403.2 |
| 56029 | 33.00 | 96.58 | 3716.9 | 56373 | 29.47 | 102.51 | 797.5 |
| 56033 | 34.55 | 98.13 | 4272.3 | 56376 | 29.20 | 102.38 | 1098.0 |
| 56034 | 33.48 | 97.08 | 4415.4 | 56380 | 29.53 | 103.21 | 571.2 |
| 56038 | 32.59 | 98.06 | 4200.0 | 56381 | 30.04 | 103.31 | 630.2 |
| 56043 | 34.29 | 100.14 | 3719.0 | 56382 | 29.45 | 103.33 | 475.0 |
| 56046 | 33.45 | 99.39 | 3967.5 | 56383 | 29.50 | 103.52 | 455.2 |
| 56065 | 34.44 | 101.36 | 3500.0 | 56384 | 29.36 | 103.29 | 446.7 |
| 56067 | 33.26 | 101.29 | 3628.5 | 56385 | 29.31 | 103.20 | 3069.9 |
| 56074 | 34.00 | 102.05 | 3471.4 | 56386 | 29.34 | 103.45 | 424.2 |
| 56079 | 33.35 | 102.58 | 3441.4 | 56387 | 29.14 | 103.16 | 642.2 |
| 56080 | 35.00 | 102.54 | 2910.0 | 56389 | 29.12 | 103.57 | 387.6 |
| 56081 | 34.42 | 103.21 | 2810.2 | 56390 | 29.40 | 104.04 | 404.4 |
| 56097 | 33.16 | 104.15 | 1440.5 | 56394 | 29.27 | 104.26 | 384.1 |
| 56106 | 31.53 | 93.47 | 4022.8 | 56459 | 27.56 | 101.16 | 2426.5 |
| 56116 | 31.25 | 95.36 | 3873.1 | 56473 | 28.57 | 102.46 | 1060.3 |
| 56125 | 32.12 | 96.28 | 3643.7 | 56474 | 28.33 | 102.10 | 1774.0 |
| 56137 | 31.09 | 97.10 | 3315.0 | 56475 | 28.39 | 102.31 | 1659.5 |
| 56144 | 31.48 | 98.35 | 3184.0 | 56478 | 28.18 | 102.26 | 1818.1 |
| 56146 | 31.37 | 100.00 | 3393.5 | 56479 | 28.00 | 102.51 | 2132.4 |
| 56147 | 31.13 | 98.50 | 3260.0 | 56480 | 28.49 | 103.32 | 564.1 |
| 56151 | 32.56 | 100.45 | 3530.0 | 56483 | 28.35 | 103.57 | 496.6 |
| 56152 | 32.17 | 100.20 | 3893.9 | 56485 | 28.16 | 103.35 | 1255.8 |
| 56158 | 31.24 | 100.40 | 3250.0 | 56487 | 28.20 | 103.08 | 1943.9 |
| 56164 | 32.16 | 100.58 | 3292.9 | 56489 | 28.14 | 103.37 | 975.6 |
| 56167 | 30.59 | 101.07 | 2957.2 | 56490 | 28.57 | 103.54 | 424.0 |
| 56168 | 31.29 | 102.04 | 2168.9 | 56533 | 27.45 | 98.40 | 1583.3 |

| | | | | | | | |
|---|---|---|---|---|---|---|---|
| 56171 | 32.54 | 101.42 | 3275.1 | 56543 | 27.51 | 99.45 | 3341.5 |
| 56172 | 31.54 | 102.14 | 2664.4 | 56548 | 27.10 | 99.17 | 2326.1 |
| 56173 | 32.48 | 102.33 | 3491.6 | 56565 | 27.26 | 101.29 | 2516.8 |
| 56178 | 31.00 | 102.21 | 2438.0 | 56569 | 27.25 | 102.11 | 1380.0 |
| 56180 | 31.41 | 103.51 | 1590.1 | 56571 | 27.54 | 102.16 | 1590.9 |
| 56182 | 32.40 | 103.36 | 2881.3 | 56575 | 27.22 | 102.33 | 1530.7 |
| 56183 | 31.30 | 103.37 | 1370.1 | 56578 | 27.04 | 102.45 | 993.6 |
| 56185 | 32.05 | 102.59 | 2400.1 | 56580 | 27.43 | 102.48 | 2460.0 |
| 56202 | 30.40 | 93.17 | 4488.8 | 56584 | 27.42 | 103.15 | 1093.0 |
| 56227 | 29.52 | 95.46 | 2736.0 | 56641 | 26.54 | 98.52 | 1189.7 |
| 56247 | 30.00 | 99.06 | 2589.2 | 56645 | 26.25 | 99.25 | 2344.9 |
| 56251 | 30.56 | 100.19 | 3000.0 | 56649 | 26.06 | 99.58 | 2059.9 |
| 56263 | 30.53 | 101.53 | 1949.7 | 56651 | 26.51 | 100.13 | 2380.9 |
| 56267 | 30.02 | 101.01 | 2600.9 | 56652 | 26.41 | 100.45 | 2130.5 |
| 56312 | 29.40 | 94.20 | 2991.8 | 56654 | 26.35 | 100.11 | 2195.4 |
| 56357 | 29.03 | 100.18 | 3727.7 | 56664 | 26.38 | 101.16 | 1230.8 |
| 56374 | 30.03 | 101.58 | 2615.7 | 56665 | 26.41 | 101.51 | 1140.3 |
| 56378 | 29.14 | 102.21 | 890.7 | 56670 | 26.55 | 102.07 | 1136.9 |
| 56434 | 28.39 | 97.28 | 2327.6 | 56671 | 26.39 | 102.15 | 1787.3 |
| 56443 | 28.56 | 99.48 | 2842.0 | 56739 | 24.59 | 98.30 | 1695.9 |
| 56444 | 28.29 | 98.54 | 3319.0 | 56742 | 25.53 | 99.22 | 1723.9 |
| 56462 | 29.00 | 101.30 | 2925.0 | 56745 | 25.41 | 99.57 | 1626.1 |
| 56567 | 27.15 | 100.51 | 2367.2 | 56746 | 25.28 | 99.31 | 1616.7 |
| 51704 | 39.43 | 76.10 | 1298.7 | 56748 | 25.07 | 99.11 | 1652.2 |
| 51705 | 39.43 | 75.15 | 2175.7 | 56751 | 25.42 | 100.11 | 1977.7 |
| 51707 | 39.30 | 76.47 | 1204.7 | 56752 | 25.50 | 100.34 | 1438.7 |
| 51708 | 39.09 | 75.57 | 1325.1 | 56835 | 24.10 | 97.47 | 959.1 |
| 51709 | 39.29 | 75.45 | 1385.6 | 56836 | 24.42 | 97.57 | 826.7 |
| 51717 | 39.15 | 76.47 | 1206.3 | 56838 | 24.00 | 97.51 | 762.9 |
| 51777 | 39.02 | 88.10 | 887.7 | 56840 | 24.49 | 98.18 | 1012.9 |
| 51802 | 38.56 | 76.10 | 1297.5 | 56841 | 24.36 | 98.41 | 1528.0 |
| 51810 | 38.55 | 77.38 | 1178.2 | 56842 | 24.44 | 99.11 | 1471.2 |
| 51811 | 38.26 | 77.16 | 1231.2 | 56844 | 24.26 | 98.35 | 913.8 |
| 51814 | 37.55 | 77.24 | 1360.4 | 57001 | 34.45 | 105.2 | 1271.9 |
| 51815 | 38.12 | 77.16 | 1274.7 | 57002 | 34.51 | 105.39 | 1216.1 |
| 51818 | 37.37 | 78.17 | 1375.4 | 57004 | 34.44 | 104.53 | 1495.4 |
| 51826 | 37.01 | 80.48 | 1336.5 | 57006 | 34.34 | 105.45 | 1149.8 |
| 51827 | 37.10 | 79.38 | 1348.9 | 57007 | 34.11 | 105.11 | 1404.6 |
| 51828 | 37.08 | 79.56 | 1375.0 | 57008 | 34.02 | 105.18 | 1579.0 |
| 51829 | 37.03 | 80.14 | 1295.6 | 57014 | 34.34 | 105.52 | 1085.2 |
| 51839 | 37.04 | 82.43 | 1409.5 | 57102 | 33.45 | 105.43 | 970.0 |
| 51855 | 38.09 | 85.33 | 1247.2 | 57105 | 33.20 | 105.36 | 1221.2 |
| 51931 | 36.51 | 81.39 | 1422.0 | 57106 | 33.19 | 106.09 | 794.2 |
| 52418 | 40.09 | 94.41 | 1139.0 | 57110 | 33.47 | 106.05 | 930.8 |

| | | | | | | | |
|---|---|---|---|---|---|---|---|
| 52424 | 40.32 | 95.47 | 1170.9 | 57204 | 32.34 | 105.13 | 782.0 |
| 52436 | 40.16 | 97.02 | 1526.0 | 57206 | 32.25 | 105.54 | 545.4 |
| 52446 | 40.18 | 99.31 | 1177.4 | 57208 | 32.17 | 105.31 | 544.5 |
| 52447 | 40.00 | 98.53 | 1270.5 | 57304 | 31.40 | 105.10 | 516.5 |
| 52546 | 39.22 | 99.50 | 1332.2 | 57307 | 31.06 | 105.05 | 459.9 |
| 52557 | 39.09 | 100.10 | 1453.7 | 57308 | 31.13 | 105.23 | 421.3 |
| 52575 | 39.25 | 102.47 | 1239.5 | 57401 | 30.52 | 105.22 | 383.3 |
| 52576 | 39.13 | 101.41 | 1510.1 | 57407 | 30.17 | 105.02 | 462.6 |

Table S2. The performance of PenPan model for monthly $E_{pan}$ at 274 stations in the QTP.

| Metrics | Minima | Maxima | Mean ± 1s.d. | Station proportion |
|---|---|---|---|---|
| Slope | 0.72 | 0.99 | 0.92 ± 0.05 | |
| $R^2$ | 0.85 | 0.99 | 0.96 ± 0.03 | 100% |
| MD (mm) | -0.40 | 0.00 | 0.00 ± 0.03 | 0 |
| RMSE (mm) | 6.68 | 41.48 | 15.92 ± 5.11 | |

Table S3. Climate zones of China.

| No | Major Climate Zone | Minor Climate Zone Code | Name |
|---|---|---|---|
| 1 | I North Temperate Zone | IA1 | Genhe Zone |
| 2 | II Mid-temperate Zone | **IIC5** | **Yining Zone** |
| | | IIC3 | Fuyun Zone |
| | | IIC4 | Tacheng Zone |
| | | IIC1 | Mengdong Zone |
| | | **IIC2** | **Mengzhong Zone** |
| | | IIA1 | Xiaoxinganling Zone |
| | | IIB1 | Daxinganling Zone |
| | | **IID1** | **Menggan Zone** |
| | | IID2 | Beijiang Zone |
| | | IIA2 | Sanjiang-Changbai Zone |
| | | IIB2 | Songliao Zone |
| 3 | III South Temperate Zone | **IIID1** | **Nanjiang Zone** |
| | | IIIC1 | Jinshangan Zone |
| | | IIIB1 | Hebei Zone |
| | | **IIIB3** | **Weihe Zone** |
| | | IIIA1 | Liaodong-Jiaodongbandao Zone |
| | | IIIB2 | Lvhuai Zone |
| 4 | IV North Subtropical Zone | **IVA2** | **Qinba Zone** |
| | | IVA1 | Jiangbei Zone |
| 5 | V Mid-subtropical Zone | VA2 | Oujiang, Minjiang, Nanling Zone |
| | | **VA1** | **Jiangnan Zone** |
| | | **VA3** | **Sichuan Zone** |
| | | VA4 | Guizhou zone |
| | | **VB1** | **Jinshajiang-Chuxiong-Yuxi Zone** |
| | | **VA5** | **Dianbei Zone** |
| 6 | VI South Subtropical Zone | VIA1 | Taibei Zone |
| | | VIA2 | Minnan-Zhujiang Zone |
| | | **VIA3** | **Diannan Zone** |
| 7 | VII North Subtropical Zone | VIIA2 | Leiqiong Zone |
| | | VIIA1 | Tainan Zone |
| | | VIIB1 | Qiongxi Zone |
| | | VIIA3 | Diannanhegu Zone |
| | | VIIC1 | Yuanjiang Zone |
| | | VIIA1 | Tainan Zone |
| 8 | VIII Mid-tropical Zone | VIIIA1 | Qiongnan-Xisha Zone |
| 9 | IX South Tropical Zone | IXA1 | Nansha Zone |
| 10 | H Highland Climate | **HD2** | **Zangbei Zone** |

| | | | |
|---|---|---|---|
| | Zone | **HC3** | **Zangnan Zone** |
| | | **HC2** | **Zangzhong Zone** |
| | | **HB2** | **Changdu Zone** |
| | | HA1 | Bomi-Chuanxi Zone |
| | | **HVVIVIIA1** | **Dawang-Chayu Zone** |
| | | **HC1** | **Qilian-Qinghaihu Zone** |
| | | HB1 | Qingnan Zone |
| | | **HD1** | **Chaidamu zone** |

Note: Climate zones used in this study were in bold.
